# Random Network and Non-rich-club Organization Tendency in Children With Non-syndromic Cleft Lip and Palate After Articulation Rehabilitation: A Diffusion Study

**DOI:** 10.3389/fneur.2022.790607

**Published:** 2022-02-02

**Authors:** Bo Rao, Hua Cheng, Haibo Xu, Yun Peng

**Affiliations:** ^1^Department of Radiology, Zhongnan Hospital of Wuhan University, Wuhan University, Wuhan, China; ^2^Department of Radiology, National Center for Children's Health, Beijing Children's Hospital, Capital Medical University, Beijing, China

**Keywords:** non-syndromic cleft lip and palate, articulation rehabilitation, diffusion tensor imaging, small-worldness, rich-club organization, graph theory

## Abstract

**Objective:**

The neuroimaging pattern in brain networks after articulation rehabilitation can be detected using graph theory and multivariate pattern analysis (MVPA). In this study, we hypothesized that the characteristics of the topology pattern of brain structural network in articulation-rehabilitated children with non-syndromic cleft lip and palate (NSCLP) were similar to that in healthy comparisons.

**Methods:**

A total of 28 children with NSCLP and 28 controls with typical development were scanned for diffusion tensor imaging on a 3T MRI scanner. Structural networks were constructed, and their topological properties were obtained. Besides, the Chinese language clear degree scale (CLCDS) scores were used for correlation analysis with topological features in patients with NSCLP.

**Results:**

The NSCLP group showed a similar rich-club connection pattern, but decreased small-world index, normalized rich-club coefficient, and increased connectivity strength of connections compared to controls. The univariate and multivariate patterns of the structural network in articulation-rehabilitated children were primarily in the feeder and local connections, covering sensorimotor, visual, frontoparietal, default mode, salience, and language networks, and orbitofrontal cortex. In addition, the connections that were significantly correlated with the CLCDS scores, as well as the weighted regions for classification, were chiefly distributed in the dorsal and ventral stream associated with the language networks of the non-dominant hemisphere.

**Conclusion:**

The average level rich-club connection pattern and the compensatory of the feeder and local connections mainly covering language networks may be related to the CLCDS in articulation-rehabilitated children with NSCLP. However, the patterns of small-world and rich-club structural organization in the articulation-rehabilitated children exhibited a random network and non-rich-club organization tendency. These findings enhanced the understanding of neuroimaging patterns in children with NSCLP after articulation rehabilitation.

## Introduction

Cleft lip and palate (CLP) is one of the most common craniofacial malformations in infants, and its prevalence has been estimated to be one in 1,000 live births (1). Non-syndromic CLP (NSCLP) is not included in any kind of well-known congenital syndrome and its incidence of articulation disorders ranges from 22 to 92% (2). Even with early surgical treatment, 30–50% of patients with CLP still suffer from cleft palate articulation characterized by hypernasality and/or nasal emissions (3). Articulation therapy is the primary management approach, along with physical management through surgery, combined with motor learning principles by visual, auditory, and touch feedback assistance (4).

Previous studies have found functional and structural changes in the brain in CLP children. The broadly decreased volumes of the subcortical nuclei and the frontal lobe (5), cerebral white matter (6), cerebellum (7), and ventral frontal cortex (8) were found in CLP children. Moreover, adult NSCLP participants after articulation rehabilitation showed increased cortical folding in brain areas related to language, auditory, and execution compared to adults with NSCLP and control participants (9). A subvocalization task performed by adults with CLP during a functional MRI study, although the left hippocampus exhibited increased activation, the rest of the brain regions showed similar brain activity in articulation rehabilitated patients relative to healthy comparison (10). Our recent study based on resting-state functional MRI that detected the increased capability of information transmission and integration involving language and social cognition brain areas in children with CLP after articulation rehabilitation. In addition, we found an increased small-world index in these patients which means optimum equilibrium between local specialization and global integration to process information (11). The structural organization of CLP children in the developing brain after articulation rehabilitation still needs to be investigated.

Graph theory has been broadly used in neuroimaging studies for healthy participants and clinical patients to assess the topological properties of network organizations (12). The graph theory technique can evaluate the small-world properties and rich-club organization of brain networks. There is an optimal balance between global integration and local specialization for information processing in a small-world brain network (13). Rich-club organization is the central hub of a network, exhibiting more density connections than the peripheral regions (14) and can promote neural signaling and integrated information across different brain areas (15). The multivariate pattern analysis (MVPA) is based on a data-driven technique widely used to analyze neuroimaging data. Such a technique could provide not only discriminative spatial patterns but also the quantification of therapy effects at the individual level (16). Additionally, the features contributing most strongly to individual classification could be identified (17). To date, there have been no MVPA studies on CLP children. Therefore, we used the MVPA to explore the structural topology pattern in articulation-rehabilitated children with NSCLP, which might improve our understanding of the recovery mechanism of structural brain networks. We hypothesized the topology pattern of brain structural network in articulation-rehabilitated childrenwith NSCLP was similar to that in healthy comparisons by the MVPA method.

In this study, diffusion tensor imaging (DTI) data were collected and structural brain networks were reconstructed for children with NSCLP after articulation rehabilitation and healthy comparison. We aimed to investigate the pattern of the structural network topology in NSCLP children after articulation rehabilitation with graph theory and the MVPA technique.

## Materials and Methods

### Participants

This study was approved by the Beijing Children's Hospital Ethical Committee and we obtained the informed consent of all the participants. A total of 28 children (mean age 10.0 ± 2.3 years, 21/7 male/female) with NSCLP and 28 typical developing healthy comparisons (age- and sex-matched) were included in this study from January 2016 to September 2017. No NSCLP participants were excluded because of the well-matching. The children with CLP would be excluded when the experienced medical geneticist suspected them within a well-known congenital syndrome by evaluating their medical histories, clinical signs, and genetics files. All the children received a Chinese speech intelligibility test administered by three experienced speech pathologists. The inclusion criteria of children were as follows: (1) aged between 6 and 16 years old; (2) Chinese as the native language; (3) a successful CLP repair for velopharyngeal insufficiency. Speech therapy started 3–6 months after the surgery, 30 min/day, 3 times/week, and lasted for half a year till patients reached a score of 86 points of the CLCDS (full credit was 100 points), which was considered as a clear line of rehabilitation; (4) normal vision and hearing auditory brainstem response (ABR) <30 dB nHL]; (5) right-handed; and (6) the Chinese Wechsler Intelligence Scale for Children-IV (CWISC-IV) scores ≥ 90, average intelligence). The exclusion criteria were as follows: (1) articulation disorder (the CLCDS scores <86); (2) dysgnosia; (3) velopharyngeal insufficiency; (4) vision and/or hearing defects; (5) congenital disorders; (6) developmental delays; (7) other chronic health diseases; and (8) other syndromes or possible syndromes.

### Image Acquisition

All the participants were scanned on a 3 T GE MRI (750, Discovery) system for DTI and T1-weighted data. For each participant, DTI data were acquired with a single-shot echo-planar imaging (EPI) sequence: matrix: 128 × 128, time repetition (TR)/time echo (TE) = 7,000/62 ms, slice thickness = 2 mm, field of view (FOV): 256 × 256, directions = 60, b-value = 0 and 1,000 s/mm^2^, and 70 continuous axial slices. The sagittal T1-weighted data were acquired with the magnetization-prepared rapid gradient echo (MPRAGE); sequence: matrix = 256 × 256 × 164, TR/TE = 8.6/3.4 ms, slice thickness = 1 mm, FOV = 240 × 240 × 164, and FA = 12°. All the axial scans were placed parallel to the anterior-posterior commissure line.

### Preprocessing

All the DTI data were processed using a pipeline tool, PANDA software (18) based on the FSL toolbox (https://fsl.fmrib.ox.ac.uk/fsl/fslwiki/). The main procedure was as follows: (1) format conversion and quality check: conversion from DICOM into NIFTI format and the deletion of poor-quality images. (2) Brain extraction and mask estimation: removal of non-brain tissues and calculation of brain masks. (3) Cropping images and eddy current correction: cropping the redundant parts of scans and correction to remove movements and eddy distortions using FSL FDT. (4) DTI metrics computation: diffusion tensor fitting and diffusion parameter, fractional anisotropy (FA) was calculated using FSL DTIFIT. (5) The FA images were coregistered from the native space to the corresponding T1-weighted images, then tissue borders were checked after coregistration. (6) Non-linear registering of the structural images from native space to the ICBM152 template for an inverse warping transformation and check tissue border after normalization. (7) The automated anatomical labeling (AAL) atlas was applied with the inverse warping transformation to individual native space. (8) The 90 regions of the AAL template inversely transformed were warped to the FA native space of each participant through the nearest neighbor interpolation method. One brain area was considered a node in the AAL atlas. (9) With fiber assignment by a continuous tracking (FACT) algorithm, white matter pathways of each DTI dataset were reconstructed and defined as fibers or tracts with streamlined tractography (19). A streamline was terminated based on its FA value <0.1 or its turn > 45° (15). Finally, from the original 33 patients, 5 patients were discharged due to poor quality, coregistration, and normalization.

### Construction of the Brain Network

We calculated the FA-based interregional connection (FABIRC) as the average of the FA values of all contained streamlines, which formed the interregional connections. The 90 × 90 FABIRC-weighted connectivity matrices were calculated for all participants. With the GRETNA toolbox (http://www.nitrc.org/projects/gretna/) (20), the topological properties of the brain structural connectivity networks were estimated for all participants. We examined the properties of the structural brain networks and applied a series of threshold values for each graph's same number of edges. In this study, the related graph properties were assessed at threshold values from 0.05 to 0.5 (step 0.01) (21).

### Network Analysis

Graph theoretical analyses of the weighted structural networks of the patients with NSCLP and controls were estimated with routines by the GRETNA toolbox.

### Small-World Properties

The network clustering coefficient (Cp) is the average clustering coefficient of the nodes of the network, representing the network segregation of the brain. The characteristic path length (Lp) is the mean shortest path length through all the possible pairs of network nodes, describing global information integration (22). The Cp was divided by the clustering coefficient of a set of random networks (*n* = 5,000) for a normalized clustering coefficient (γ) and the normalized path length (λ) was acquired using the same method (20). Small-worldness (σ) is defined as γ divided by λ and σ > 1 for small-world networks, characterizing the equilibrium of network segregation and integration (22).

### Rich-Club Organization

The ratio of existing connections to full connections of the rich-club is defined as the rich-club coefficient (Φ) (23). Normalized rich-club coefficients (Φ_norm_) are normalized Φ by a set of 1000 comparable random networks Φ_rand_ (Φ_norm_ = Φ/Φ_rand_) (24). Φ_norm_ > 1 suggests the existence of rich-club organization across ranges of degrees (k) at a density of 16% (15, 25).

### Rich-Club Regions

Ranking the brain Regions with the averaged degree across all the groups, the top 13 (15%) nodes were selected as the rich-club regions (25, 26). Based on the rich-club and non-rich-club regions, edges of the network are classified into rich-club connections between the rich-club regions, feeder connections between the rich-club and non-rich-club regions, and local connections between the non-rich-club regions (23). In addition, we calculated the connectivity strength of the three connection classes by summing the edge weights.

### Classification of the Support Vector Machine (SVM)

We applied the *F* score method for feature selection (27). We used the leave-one-out cross-validation (LOOCV) method to estimate a classifier's performance by the limited number of samples (28). We adopted the linear kernel SVM classifier for classification using the LIBSVM toolbox, with optimized parameter Cs (27). Based on the LOOCV results, we quantified the performance of the classifier by the parameters of accuracy, sensitivity, specificity, and area under the receiver operating characteristic (ROC) curve (AUC). We used a permutation test to correct the classification results (1,000 times) (29). Furthermore, we considered the consensus features as the common features, which were always chosen for the final feature set by every LOOCV iteration. Finally, we defined the weighted regions from the consensus features. The previous literature describes the detailed steps (30).

### Statistical Analysis

The statistics module was used to carry out all the statistical analyses in the GRETNA and LIBSVM toolboxes. We calculated the AUCs of small-world parameters over the density range (0.05–0.50). We used the two-sample *t*-tests to evaluate the intergroup differences in small-world properties, rich-club coefficients, normalized rich-club coefficients, rich-club, feeder and local connections, and three classes of connectivity strengths corrected for age and sex. In addition, the Chinese language clear degree scale (CLCDS) scores were performed for correlation analysis with the significant topological properties and consensus features in NSCLP children after articulation rehabilitation.

**Figure 1:**
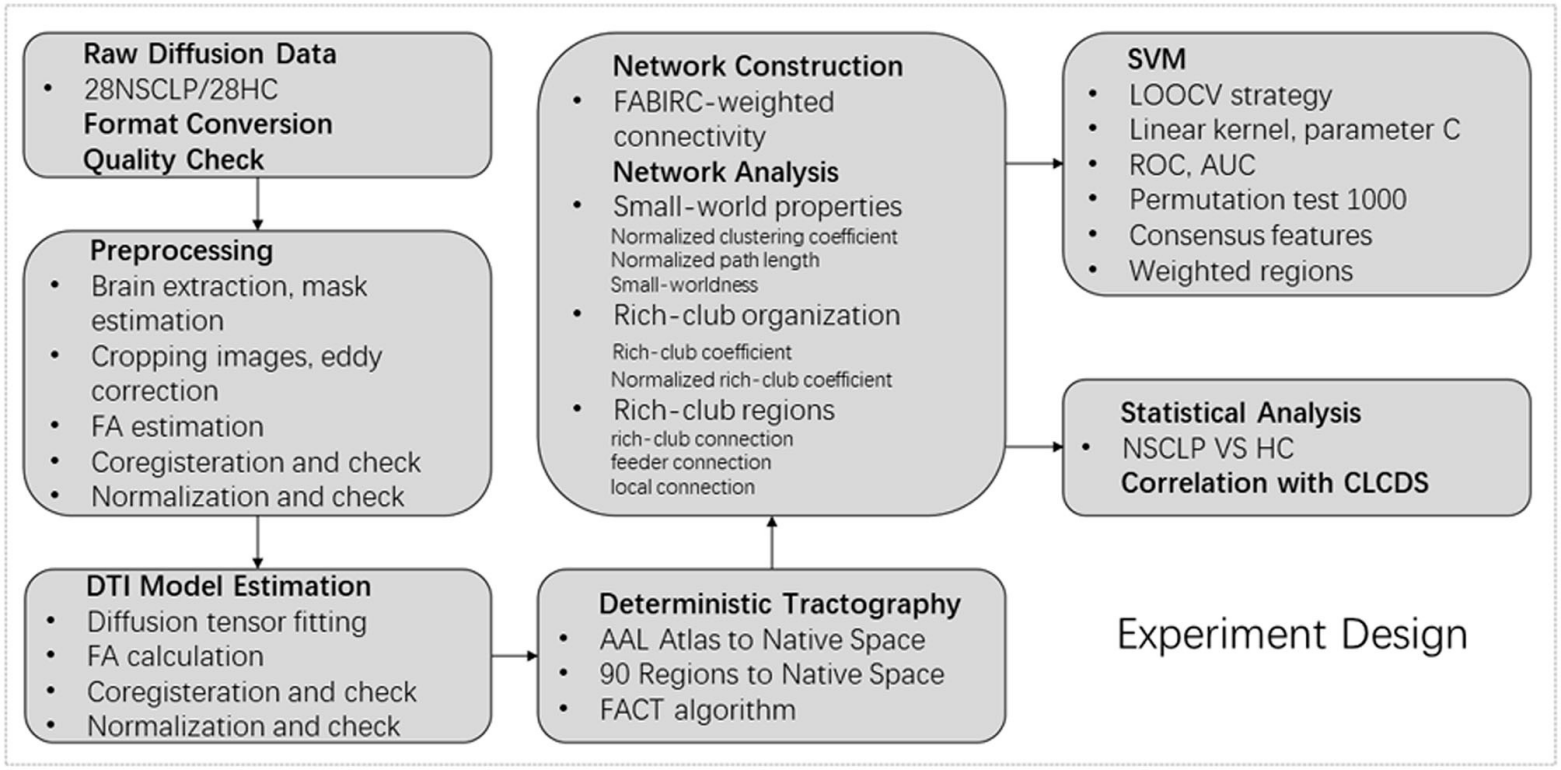
Flowchart of experiment design.

The flowchart of the experiment design is shown in Figure 1.

## Results

In this study, structural networks of the whole brain were constructed and the small-world and rich-club properties of children with NSCLP and healthy comparison were calculated in the density range of 0.05–0.50 (step 0.01).

### Demographic Characteristics

The demographic characteristics of children are shown in Table 1, Supplementary Table S1. The age of children (*t* = −0.46, *p* = 0.96), CWISC-IV scores (*t* = −1.19, *p* = 0.24), and education (*t* = −1.10, *p* = 0.28) showed no significant between-group differences. The number of males was slightly higher than that of females among children with NSCLP and healthy comparison, but the distribution showed no significant differences (chi-squared test, χ^2^ = 0, *p* = 1) (see Table 1).

**Table 1:** Demographic and clinical characteristics.

	**Age (years)**	**Boys/Girls**	**CLCDS score**	**CWISC-IV score**	**Education (years)**	
**Sample members**	**Mean** **±SD**	**Median**	**No**.	**Mean** **±SD**	**Mean** **±SD**	**Mean** **±SD**
NSCLP children	10.0 ± 2.3	9.6	21/7	91.6 ± 4.0	97.5 ± 9.5	4.0 ± 2.2
Healthy controls	10.4 ± 2.0	9.5	21/7	–	99.8 ± 6.6	4.7 ± 2.3

*NSCLP, non-syndromic cleft lip and palate; CLCDS, Chinese language clear degree scale; CWISC-IV, Chinese Wechsler Intelligence Scale for Children-IV*.

### Small-World Properties

**Figure 2:**
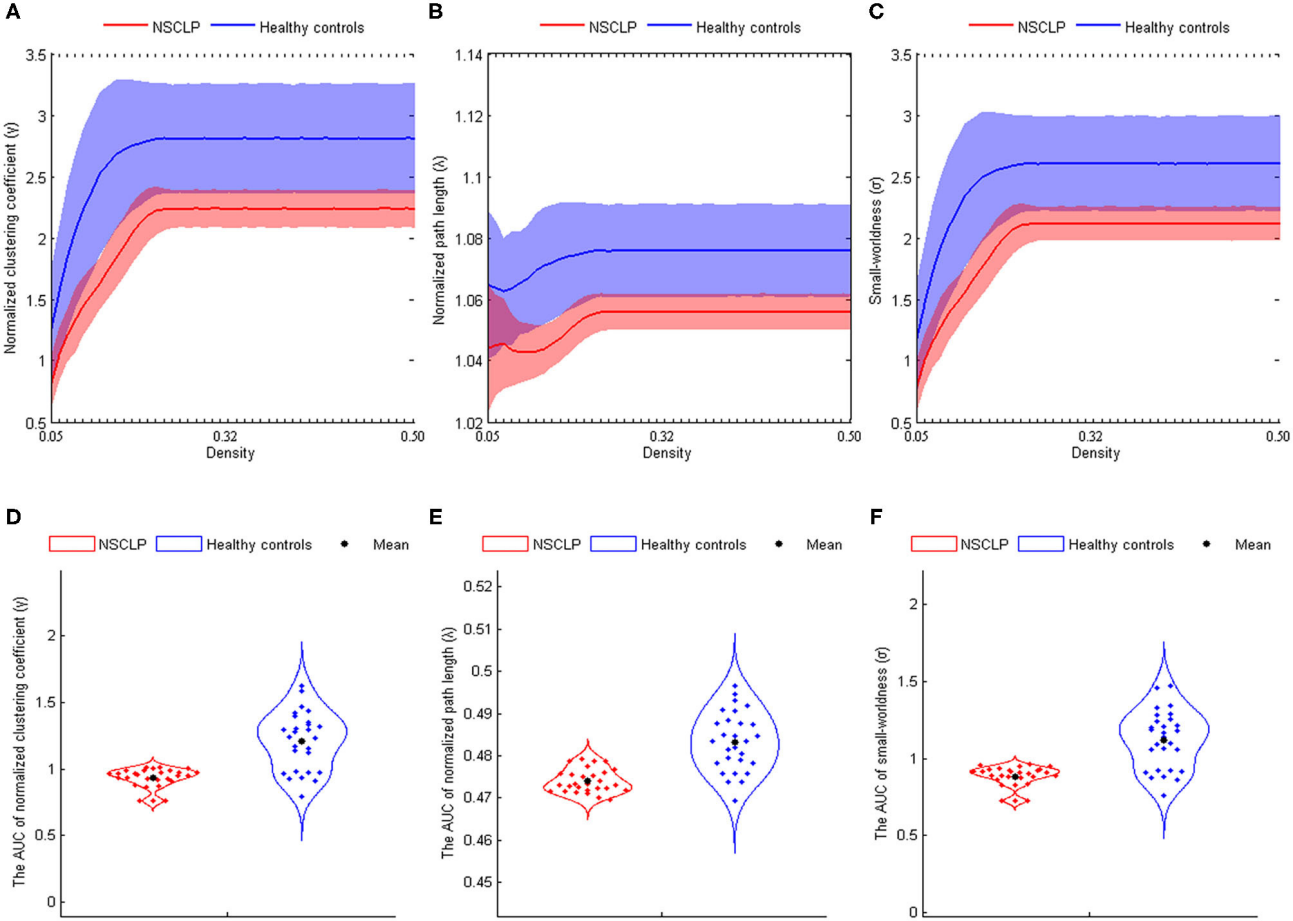
**(A–C)** The normalized clustering coefficient **(A)**, normalized path length **(B)**, and small-world index **(C)** shown at the whole threshold. Shaded areas represent the standard deviation of the mean. **(D–F)**: Significant differences in the normalized clustering coefficient **(D)**, normalized path length **(E)**, and small-world index **(F)**. Corrected for age and gender. Two-sample two-tailed *t*-test, *p* < 0.05. AUC, area under the curve; NSCLP, non-syndromic cleft lip and palate; HC, healthy comparison.

In both the groups, the structural networks showed a small-world organization in the density of 0.06–0.50 (σ > 1, see Figures 2A–C). Furthermore, there were lower σ^AUCs^ (*t* = −6.639, *p* < 0.001), γ^AUCs^ (*t* = −6.752, *p* < 0.001), and λ^AUCs^ (*t* = −7.028, *p* < 0.001) in articulation-rehabilitated patients with NSCLP (see Figures 2D,E).

### Rich-Club Organization

**Figure 3:**
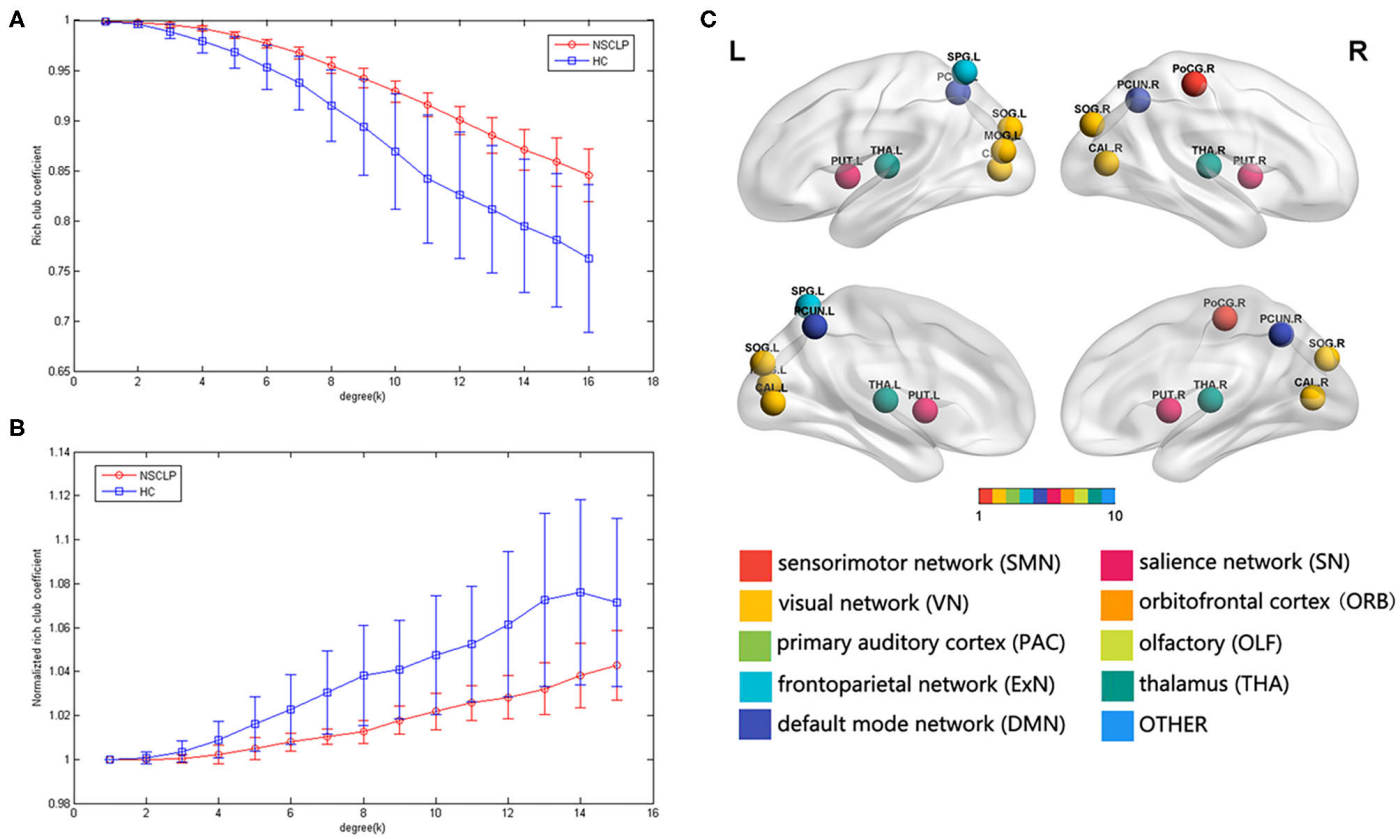
**(A,B)** Group-averaged rich-club curve and standard error for patients (red) and controls (blue). **(A)**: rich-club coefficient (Φ), **(C)**: normalized rich-club coefficients (Φ_norm_). **(C)**: rich-club regions across the patients and controls. The color of the balls indicates the intrinsic connectivity networks.

We found the rich-club organization and normalized rich-club coefficients Φ_norm_ >1 in children with NSCLP after articulation rehabilitation and the healthy comparison. A series of two-sample *t*-tests showed significant between-group differences in rich-club coefficients (*k* = 4–16, see Figure 3A, Supplementary Table S2) and normalized rich-club coefficients Φ_norm_ (*k* = 5–15, see Figure 3B, Supplementary Table S2). The rich-club effects of most participants in each group (90%) were reported. Based on the averaged nodal degree across both groups, we considered the top 13 (15%) highest-degree nodes to be rich-club regions. The rich-club regions are exhibited in **Figure 5**, including 13 areas (in order of degree): the bilateral calcarine fissure and surrounding cortex (CAL), superior occipital gyrus (SOG), superior parietal lobule, precuneus (PCUN), putamen (PUT), thalamus (THA), and left middle occipital gyrus, which is in line with previous rich-club findings (31) (see Figure 3C). The remaining 73 regions were identified as peripheral regions.

### Rich-Club Connections

**Figure 4:**
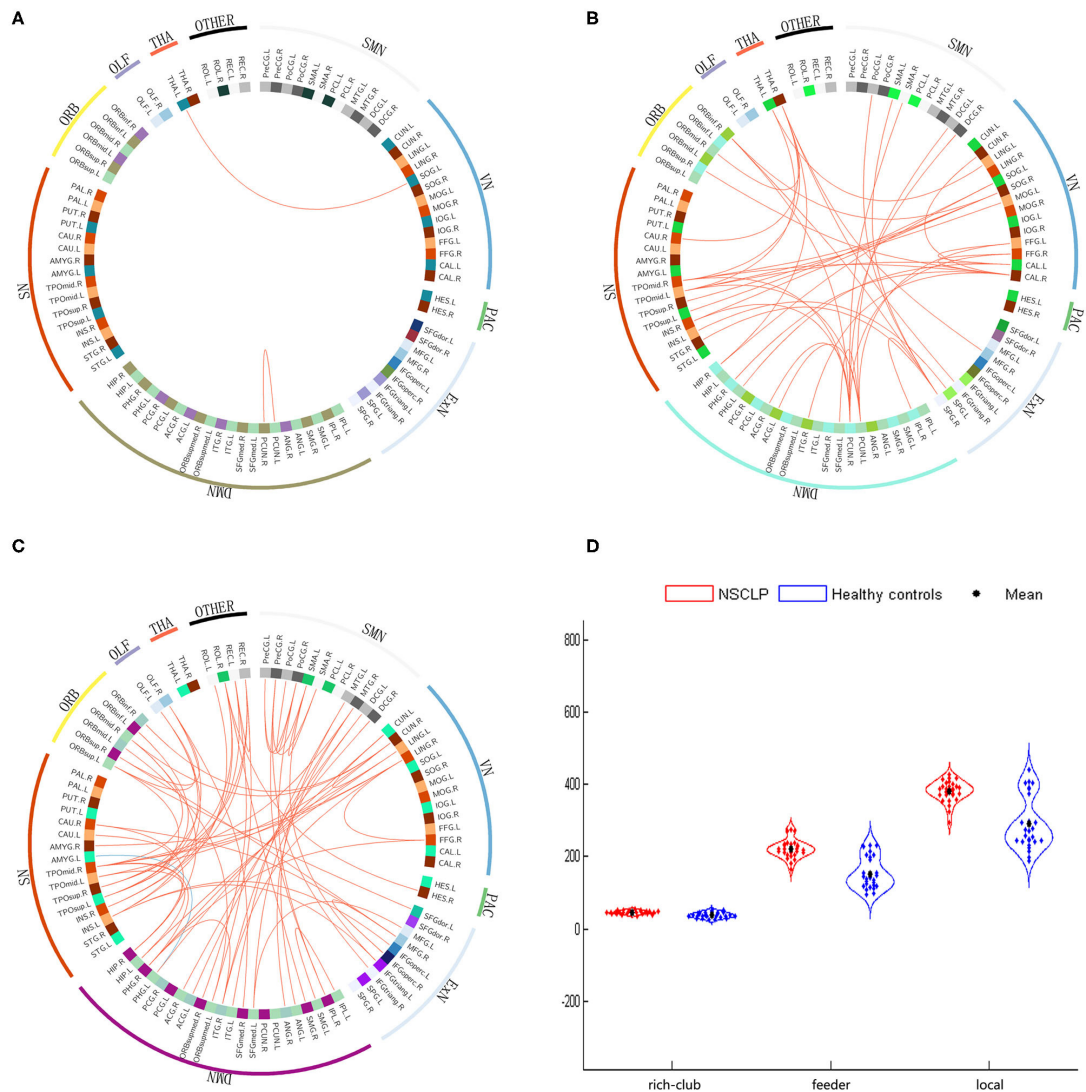
**(A–C)**: The different connections in the NSCLP group relative to healthy comparison. **(A)**: rich-club connections, **(B)**: feeder connections, **(C)**: local connections. Red edges indicate higher connectivity strength in the NSCLP group than in healthy comparison, and blue edges indicate lower connectivity strength. **(D)**: significantly altered between-group connectivity strength illustrated by rich-club, feeder, and local connections. SMN, sensorimotor network; VN, visual network; Exner, external frontoparietal network; DMN, default mode network; SN, salience network; ORB, orbitofrontal cortex; PAC, primary auditory cortex; TAH, thalamus; OLF, olfactory cortex.

We found significantly higher weighted rich-club connections between the left SOG and THA (*t* = 2.75, *p* < 0.001) and the left and right PCUN (*t* = 4.57, *p* < 0.001) in the NSCLP group than in the healthy comparison (see Figure 4A).

### Feeder Connections

We detected that the values of the 42 feeder connections in the NSCLP group were significantly higher than those in the healthy comparison (*p* < 0.001). These peripheral regions were mostly involved in the sensorimotor network (SMN), visual network (VN), external frontoparietal network (ExN), default mode network (DMN), salience network (SN), and orbitofrontal cortex (ORB) (see Figure 4B) (30, 32, 33).

### Local Connections

We observed that only one local connection between the left Parahippocampal gyrus (PHG) and amygadala nucleus (AMYG) showed a lower weighted value (*t* = −4.12, *p* < 0.001). In contrast, the other 66 local connections exhibited increased weighted values in the NSCLP children (*p* < 0.001). These peripheral regions extensively affected the SMN, VN, ExN, DMN, SN, and ORB (see Figure 4C).

### Rich-Club, Feeder, and Local Connectivity Strength

The two-sample *t*-tests were applied to test for differences in the strength of the rich-club, feeder, and local connections. We discovered that children with NSCLP showed significantly increased strength of rich-club (*t* = 4.796, *p* < 0.001), feeder (*t* = 7.324, *p* < 0.001) and local (*t* = 6.173, *p* < 0.001) connections compared to the healthy comparison (see Figure 4D).

### Classification of the Support Vector Machine (SVM)

**Figure 5:**
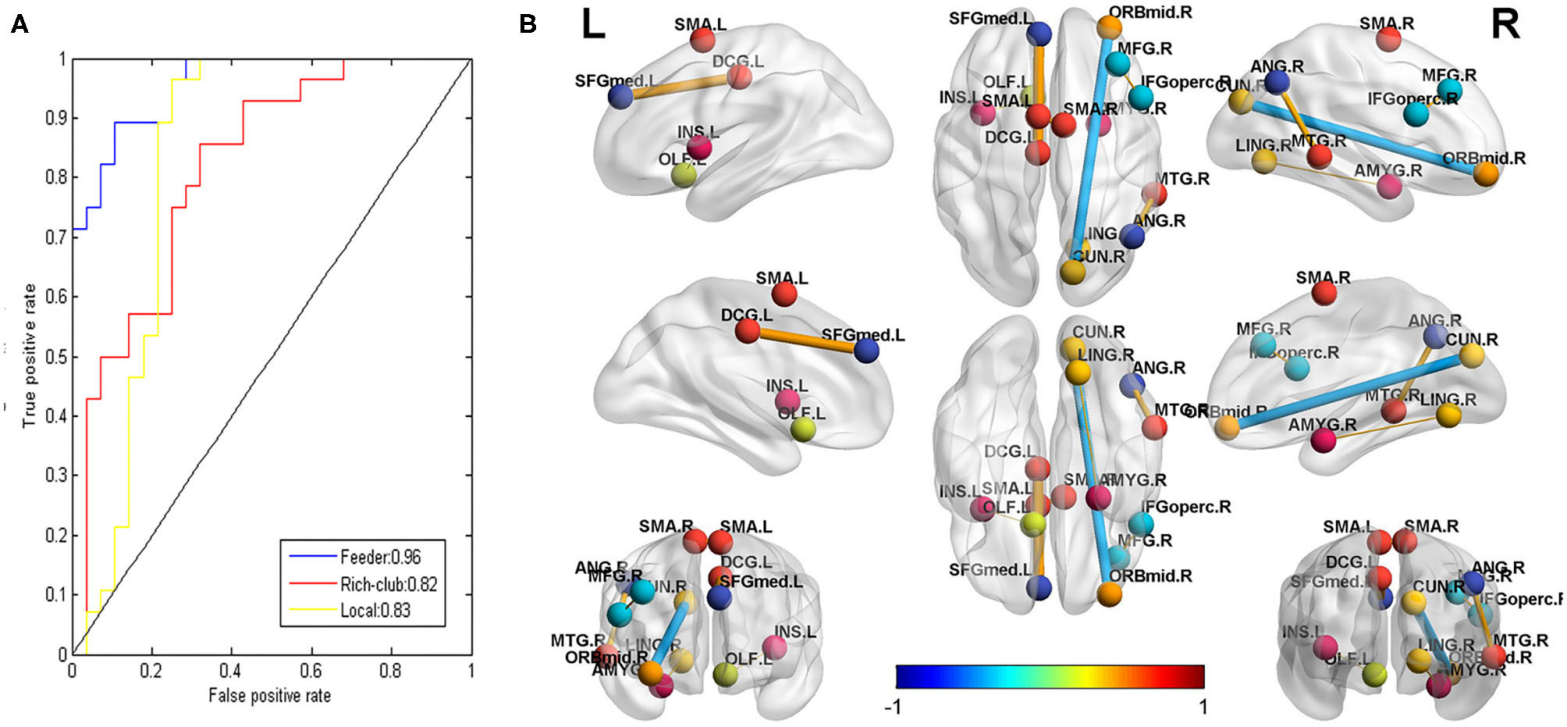
**(A)**: The receiver operating characteristic (ROC) curve of the classifier for rich-club (red, AUC = 0.82), feeder (blue, AUC = 0.96), and local (yellow, AUC = 0.83) connections. AUC: the area under the ROC curve. **(B)**: the connections that were significantly correlated with the CLCDS scores. The balls represent brain areas, and the colors of the balls indicate the intrinsic connectivity networks. The size and color of the lines (connections) represent the *r*-value and correspond to the color bar.

As shown in Supplementary Figure S1, the rich-club connections with the 28 highest ranked connections showed an accuracy of 75% (sensitivity 86%, specificity 68%, *p* < 0.05), the local connections with the 2,660 highest ranked connections showed an accuracy of 86% (sensitivity 96%, specificity 75%, *p* < 0.001), and the feeder connections with the 320 highest ranked connections showed an accuracy of 89% (sensitivity 96%, specificity 75%, *p* < 0.001) (see Figure 5A). The AUCs of the rich-club, feeder, and local connections were 0.82, 0.96, and 0.83, respectively (see Figure 5A).

**Figure 6:**
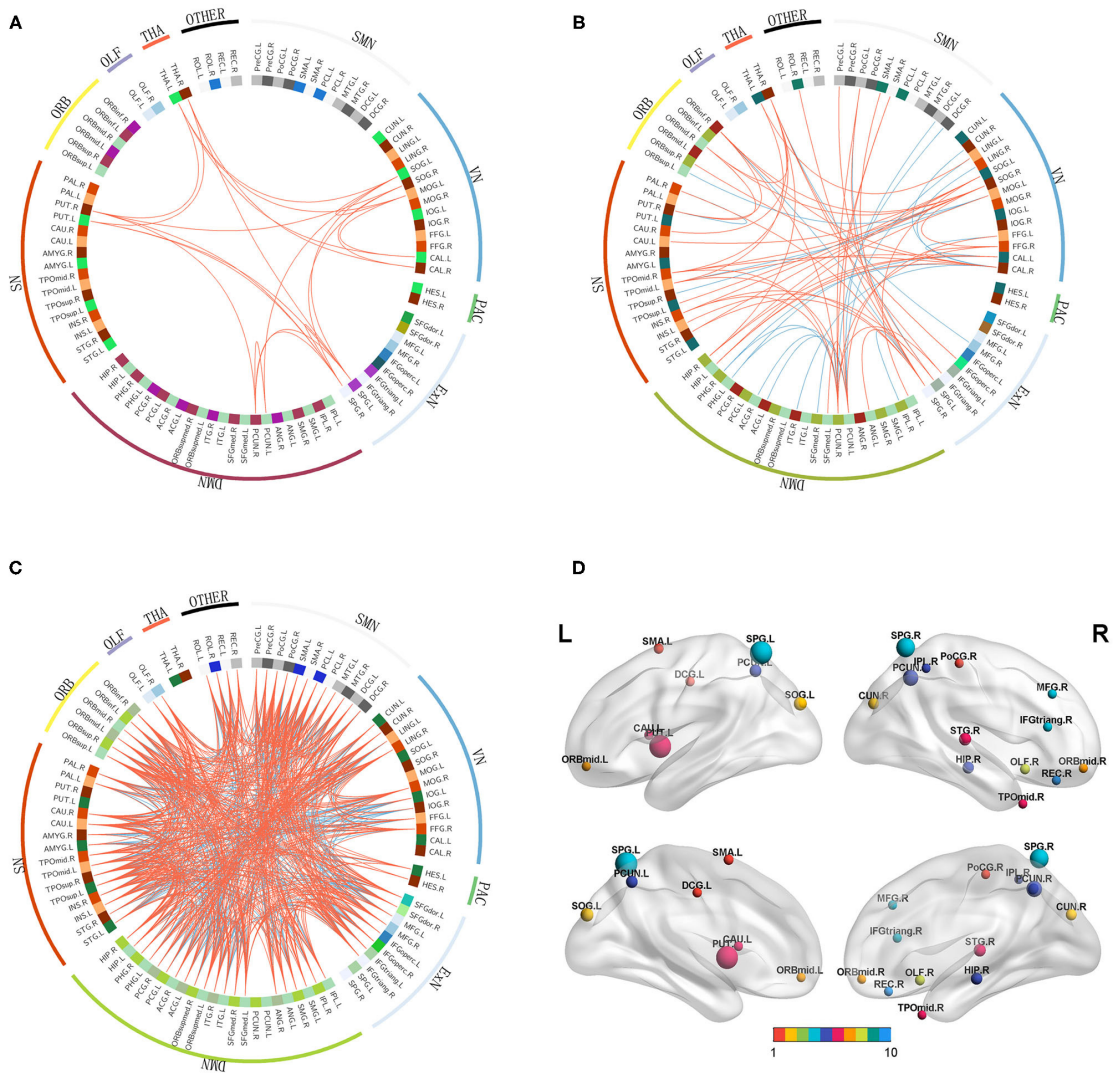
**(A–C)**: Consensus functional connections. The red lines represent positive connections, and the blue lines represent negative connections. **(D)**: The distribution of weighted regions in the NSCLP group. The size of the balls indicates the normalized classification weights, and the color of the balls represents the intrinsic connectivity networks. (1:SMN, sensorimotor network; 2:VN, visual network; 3:ExN, frontoparietal network; 4:DMN, default mode network; 5:SN, salience network; 6:ORB, orbitofrontal cortex; 7:PAC, primary auditory cortex, 8:THA, thalamus; 9:OLF, olfactory cortex; 10 other).

A total of 22 (rich-club connections), 674 (local connections), and 71 (feeder connections) consensus features were identified in the cross-validation. Consensus structural connectivity was detected primarily in the SMN, VN, ExN, DMN, SN, and ORB (see Figures 6A–C).

Among the consensus features, 2 (rich-club), 14 (local), and 6 (feeder) regions were identified as weighted regions (see Supplementary Figure S2), which had weights that were at least one standard deviation greater than the average of the weights of all areas. The three types of weighted brains were normalized (see Figure 6D) and were mainly distributed in the dorsal stream associated with phonological processing and the ventral stream related to semantic processing (34).

### Relationships Between Neuroimaging Properties and the CLCDS

Seven local connections showed a significant correlation with CLCDS and were also consensus features identified in the cross-validation. The connection between the right middle frontal gyrus (MFG), orbital part (ORBmid), and cuneus (CUN) (*r* = −0.4388, *p* = 0.0195 was a negative classification weight. The rest of the connections were positive between the right inferior frontal gyrus, opercular part (IFGoperc) and MFG; the right and left supplementary motor area (SMA), left insula, and olfactory cortex (OLF); the left superior frontal gyrus, medial (SFGmed) and median cingulate and paracingulate gyri (DCG); the right lingual gyrus (LIN) and amygdala (AMYG); and the right middle temporal gyrus (MTG) and angular gyrus (ANG), all mainly involving language-related brain regions (see Figure 5B, Supplementary Figure S3, Table 2).

**Table 2:** The connection significantly correlated with the CLCDS scores.

	**Connections (AAL atlas)**						
Brain areas	12	46	20	29	33	48	86
	8	10	19	21	23	42	66
p	0.04	0.02	0.03	0.05	0.02	0.04	0.03
r	0.39	−0.44	0.40	0.38	0.44	0.38	0.41

*The number of brain areas corresponds to the code of the automated anatomical labeling (AAL) atlas.*

*CLCDS, Chinese language clear degree scale*.

## Discussion

To the best of our knowledge, this diffusion MRI study was the first to explore the topology of white matter structural networks in children with NSCLP after articulation rehabilitation. In this study, the b-values of our diffusion data were relatively low (b = 1,000) and not suitable for the spherical deconvolution or other advanced signal representations/models (35). Multifiber deterministic fiber tract imaging (FACT) is superior to probabilistic fiber tract imaging in connectome mapping (36). Therefore, we used the tensor model and deterministic tractography for the structural network construction. First, compared with the healthy comparison, a lower σ and Φ_norm_ were detected in the structural brain networks of children with NSCLP after articulation rehabilitation. Second, the three types of connectivity strength were higher in the NSCLP group than in the healthy comparison. Additionally, the significant between-group differences in the connections were mainly located in the feeder and local connections. Third, the three types of connections showed a higher classification power, especially for the feeder connections. The consensus features were mostly in the feeder and local connections. Fourth, the local connections and consensus features were significantly correlated with the CLCDS scores, and the weighted regions were distributed in the dorsal and ventral streams related to articulation processing in the non-dominant language hemisphere.

### Small-World Properties

We detected small-worldness (σ > 1, density 0.06–0.50) in both groups, which indicated that articulation-rehabilitated children with NSCLP and healthy comparison showed parallel information transfer at a low cost and an optimal equilibrium of global integration and local specialization (density 0.06–0.50) (37). However, articulation-rehabilitated patients showed the lower AUC values of σ, γ, and λ than healthy comparison. The decreased γ meant reducing the brain's network segregation, and a lower λ represented stronger global information integration (22). The lower σ suggested a disturbance of the average balance and a higher cost for a new equilibrium between weaker local phonologic information transfer and higher global information integration, demonstrating a random network tendency in articulation-rehabilitated children with NSCLP (22). However, our previous study showed that the same participants exhibited a higher σ value of the resting-state functional network in the NSCLP group (11).

We speculated that the neuroplasticity of the structure might occur later than that of the function. The phenotype of the separation between the structural and functional networks was detected in patients with subacute intracerebral hemorrhage (38). In a rehabilitation practice of peripheral nerve-injured rats, rehabilitation therapy of electroacupuncture for 120 days can induce the neuroplasticity of the structural network, but a treatment duration of only 30 days cannot induce the neuroplasticity of the structural network (39). The results of these studies are consistent with our findings.

### Rich-Club Organization

Rich-club organization is a basis for efficient global information transfer and complex neurological function in the brain (23). We detected rich-club organizations with an increasing normalized rich-club coefficient (_norm_ > 1, *k* = 2–16) across the two groups, representing the existence of a rich-club organization in structural networks (31). Similar results were found in major depressive disorder after selective serotonin reuptake inhibitor treatment in the structural network (40). Compared with the healthy comparison, the _norm_ in the NSCLP group was significantly decreased, mostly in the low-degree regimen (*k* <16). This finding indicates that connections linked to rich-club regions decrease in the total amount of the strongest connections they could share. This result suggests that the integration of information between peripheral brain regions and their engagement in various types of cognition measured by the CLCDS scores were weaker in the NSCLP group than in healthy comparison (41). We speculated that although the CLCDS scores reached the average level, the speech errors were not completely corrected (42). The non-rich-club organization of the structural network may be the phenotype of the residual articulation errors in the NSCLP children after speech rehabilitation.

### Rich-Club, Feeder, and Local Connections

We found that the connection values in the NSCLP group were significantly higher than those in healthy comparison, mainly located in the feeder and local connections (two rich-club connections); only one connection between the left PHG and AMYG was a lower value compared to controls. A similar network pattern was seen in major depressive disorder after treatment (40). These findings indicate that the significantly different between-group connections after articulation rehabilitation are mostly in the feeder and local connections involved in all of the intrinsic connectivity networks, including the DMN, SMN, SN, VN, primary auditory cortex, ORB, and ExN (30, 32, 33). The special patterns of rich-club organization in NSCLP children after articulation rehabilitation can provide us with a new perspective.

We identified a similar rich-club connection pattern, except two higher strength connections (the left SOG and THA, left PCUN and right PCUN) in articulation-rehabilitated children with NSCLP compared with healthy comparisons. Our results indicated that efficient global information transfer and complex neurological function nearly reached the average level (15), representing average level CLCDS scores. The THA serves as a relay station and applies modulation (43). The SOG and PCUN play a central role in a wide spectrum of highly integrated tasks, including visuospatial imagery, episodic memory retrieval, and self-processing operations (44). We speculated that articulation-rehabilitated NSCLP children might balance residual articulation defectiveness through the increased compensatory function of the modulation and integrated tasks for global information integration of the rehabilitated cognitive functions (CLCDS scores > 90). This result is similar to a subvocalization task functional MRI study in adults after articulation rehabilitation, exhibiting only left hippocampal activation (10).

We detected that the 42 feeder and 66 local connections in the NSCLP group were significantly higher than those in the healthy comparison, covering the peripheral regions involved in the DMN, SMN, SN, VN, PAC, ORB, and ExN. Our results indicated that an increase in structural connectivities induced by speech therapy is mainly located in the peripheral regions. Therefore, the cost of the structural network was increased, which is consistent with the small-worldness alterations of this study. Neuroimaging studies have found that the brain undergoes remodeling induced by short-term training, exhibiting white matter restructuring (45, 46). We speculated that because the NSCLP group imitated the articulation of instructors by visual, auditory, and touch feedback assistance and the white matter connectivities between the affected peripheral regions produced neuroplasticity in the DMN, SMN, SN, VN, PAC, ORB, and ExN.

Only the connection between the left PHG and AMYG was lower in the NSCLP group than in the healthy comparison. The PHG plays a critical role in forming pathological memories (47), and the AMYG is emotionally associated with decision-making (48). This finding suggested that decision-making memory of articulation was impaired, which may be induced by the residual speech errors in the NSCLP group. A pattern of PHG was identified in internet gaming disorder in a resting-state fMRI study (49), which supports our findings.

### Connectivity Strength

We found that the rich-club, feeder, and local connection strength were significantly higher in the NSCLP group than in the healthy comparison. Rich-club organization is associated with cognitive performance (50). Our results indicated that the strengths of the rich-club, feeder, and local connections were stronger in the NSCLP group. We conjectured that the stronger connectivity strength might compensate for residual articulation defects measured by the CLCDS scores (42).

### SVM Classification

We employed the *F*-score for the feature ranking in the feature selection approach, and an SVM algorithm with a LOOCV strategy showed classification accuracies (75, 86, and 89%) and AUCs (0.82, 0.96, and 0.83) for the rich-club, feeder, and local connections, respectively. Our results indicated that the three types of connections showed good classification ability, especially the feeder connections. Simultaneously, the results suggested that the structural connection pattern in the NSLCP group was specific and different from that in the healthy comparison. We speculated that speech therapy might induce neuroplasticity changes in these connections to improve language function as measured by the CLCDS scores in the NSCLP group, especially the feeder connections. The feeder connections of the structural network showed the most discriminative power in major depressive disorder after treatment (40), which was consistent with our findings.

### Consensus Features

The distribution of consensus features was similar to the univariate analysis in the three types of connections, widely involved in the SMN, VN, ExN, DMN, SN, and ORB, which was consistent with a study of the treatment in a major depressive disorder (40). These brain networks are engaged in sensorimotor function, visual processing, executive functioning, auditory processing, language, memory, attention, and/or even consciousness (51, 52). The ORB links subjective values to valuation and choice (53). Our results indicated that articulation rehabilitation resulted from a combination of multiple intrinsic connectivity networks and the cortex. During the speech rehabilitation procedure, the NSCLP group had to comprehend and learn the articulation model, imitate the doctor, and keep repeating speech to achieve a nearly normal vocalization. Therefore, the brain regions associated with sensorimotor, language, visual, auditory, and executive functioning were intensely involved (9).

### Weighted Regions

We identified the weighted regions that contributed the most to the accurate classification. These regions were mainly distributed in the dorsal stream associated with phonological processing and in the ventral stream related to semantic processing (34, 54, 55), except for the left SOG, PUT, caudate nucleus (CAU), DCG, right CUN, hippocampus (HIP), and OLF. The CUN and SOG are involved in visual processing (56) and integrate the somatosensory information and cognitive processes such as attention, learning, and memory (57). The HIP is related to memory. The PUT and CAU are linked with motor skills and neurofeedback learning (58, 59). The DCG is the key region for proactive rather than reactive action control, indicated by increased neural activity for endogenous action selection (60). Our results showed that the weighted regions were more abundant in the right hemisphere, which suggested that articulation rehabilitation mainly induced neuroplasticity in the brain regions involved in language, memory, vision, and active learning skills in the non-dominant language hemisphere. Because the impaired brain regions in children with NSCLP with speech therapy were mainly located in the language dominant hemisphere (6), we speculated that the relatively intact non-dominant language hemisphere provided a compensatory function for articulation rehabilitation.

### Relationship Between the Connections and the CLCDS

We detected seven local connections, which were also the local connections' consensus features, and showed significant correlations with the CLCDS scores. The brain regions involved in the seven connections showed a similar distribution to the weighted regions, mainly in the dorsal and ventral stream associated with language in the right hemisphere. The language-related connections were between the right MFG and IFGoper, ANG and MTG, LING and AMYG, the left SFG and DCG, left INS and OLF, and the left and right SMA (34, 54, 55). The only negative connection was the one between the right CUN and ORBmid. This finding is similar to that found in a study of stutterers (61). As mentioned above, the CUN is involved in vision, and the ORB links subjective values to valuation and choice (53). We speculated that the increased connectivity of the dorsal and ventral streams caused by the articulation train might promote phonological and semantic processing. The negative correlation with the CLCDS scores implies that the clear articulation may not need to be regulated by high-order brain regions.

### Limitations

There were several limitations in this study. First, the sample size in this study was relatively small. Second, more study focused on investigating the structural, topological properties in untreated or non-rehabilitated children with NSCLP should be conducted in the future. Third, multikernel learning based on multimodal features should be applied to a larger sample. Fourth, with age, whether these topological parameters will return to normal levels or complete speech correction is required in further research.

## Conclusion

The feeder and local connections involving SMN, VN, ExN, DMN, SN, and ORB may perform a compensatory function during articulation rehabilitation. In addition, the language networks may imply a vital role of language processing in articulation rehabilitation. However, articulation-rehabilitated children with NSCLP exhibited a random network and non-rich-club organization tendency. These findings provide a profound understanding of neuroimaging in children with NSCLP after articulation rehabilitation.

## Data Availability Statement

The original contributions presented in the study are included in the article/Supplementary Material, further inquiries can be directed to the corresponding authors.

## Ethics Statement

The studies involving human participants were reviewed and approved by Beijing Children's Hospital Ethical Committee. Written informed consent to participate in this study was provided by the participants' legal guardian/next of kin.

## Author Contributions

HC contributed to methodology, software, formal analysis, writing—original draft, and investigation. BR contributed to writing—review and editing, project administration, validation, and data curation. HX contributed to resources and supervision. YP contributed to funding acquisition. All authors contributed to the article and approved the submitted version.

## Funding

This study was supported in part by the National Natural Science Foundation of China (Grant No. 81671651), the Yang Fan Plan of Beijing Municipal Administration of Hospital Clinical Innovation Project, (Grant No. XMLX201714), and the Cultivate Plan of Beijing Municipal Administration of Hospital (Grant No. PX2018047).

## Conflict of Interest

The authors declare that the research was conducted in the absence of any commercial or financial relationships that could be construed as a potential conflict of interest.

## Publisher's Note

All claims expressed in this article are solely those of the authors and do not necessarily represent those of their affiliated organizations, or those of the publisher, the editors and the reviewers. Any product that may be evaluated in this article, or claim that may be made by its manufacturer, is not guaranteed or endorsed by the publisher.
