# Supplementary material for: Random Network and Non-rich-club Organization Tendency in Children With Non-syndromic Cleft Lip and Palate After Articulation Rehabilitation: A Diffusion Study
Source: Front Neurol. 2022 Feb 2;13:790607. doi: 10.3389/fneur.2022.790607 (PMC8847279; doi:10.3389/fneur.2022.790607)
Supplement: Supplementary file 1 [file Data_Sheet_1.zip › supplement/supplement.docx]

Table S1 Detail demographic and clinical characteristics

| NSCLP | | | | | |  | HC | | |
| --- | --- | --- | --- | --- | --- | --- | --- | --- | --- |
| code | sex | age (year) | age at surgery | duration (year) | type |  | code | sex | age (year) |
| sub01 | 1 | 10.8 | 10.3 | 0.5 | CLP |  | sub029 | 2 | 13.6 |
| sub02 | 2 | 15.0 | 14.6 | 0.4 | CLP |  | sub030 | 1 | 9.2 |
| sub03 | 1 | 8.8 | 8.5 | 0.5 | CP |  | sub031 | 1 | 14.5 |
| sub04 | 1 | 7.7 | 7.3 | 0.5 | CLP |  | sub032 | 1 | 13.5 |
| sub05 | 1 | 9.5 | 9.2 | 0.4 | CLP |  | sub033 | 2 | 9.2 |
| sub06 | 1 | 7.2 | 6.9 | 0.5 | CLP |  | sub034 | 1 | 9.1 |
| sub07 | 2 | 8.2 | 7.9 | 0.3 | CLP |  | sub035 | 2 | 9.3 |
| sub08 | 2 | 7.6 | 7.3 | 0.3 | CLP |  | sub036 | 1 | 13.4 |
| sub09 | 1 | 9.5 | 9.2 | 0.5 | CP |  | sub037 | 1 | 11.6 |
| sub10 | 1 | 9.2 | 8.9 | 0.5 | CP |  | sub038 | 1 | 11.7 |
| sub11 | 1 | 15.4 | 15.1 | 0.4 | CLP |  | sub039 | 1 | 12.3 |
| sub12 | 1 | 12.6 | 12.3 | 0.3 | CLP |  | sub040 | 2 | 8.1 |
| sub13 | 1 | 11.7 | 11.2 | 0.5 | CLP |  | sub041 | 2 | 8.9 |
| sub14 | 1 | 9.4 | 8.9 | 0.4 | CLP |  | sub042 | 2 | 9.1 |
| sub15 | 1 | 10.4 | 10.0 | 0.3 | CLP |  | sub043 | 1 | 10.8 |
| sub16 | 1 | 10.5 | 10.1 | 0.4 | CLP |  | sub044 | 1 | 11.1 |
| sub17 | 1 | 7.6 | 7.3 | 0.4 | CLP |  | sub045 | 1 | 12.6 |
| sub18 | 1 | 9.8 | 9.3 | 0.5 | CLP |  | sub046 | 1 | 6.9 |
| sub19 | 1 | 9.3 | 9.0 | 0.4 | CLP |  | sub047 | 1 | 10.5 |
| sub20 | 1 | 10.0 | 9.7 | 0.5 | CLP |  | sub048 | 1 | 10.3 |
| sub21 | 1 | 12.5 | 12.1 | 0.4 | CP |  | sub049 | 1 | 9.9 |
| sub22 | 1 | 11.5 | 11.2 | 0.5 | CLP |  | sub050 | 2 | 7.0 |
| sub23 | 1 | 13.0 | 12.7 | 0.4 | CLP |  | sub051 | 1 | 9.5 |
| sub24 | 1 | 7.2 | 6.9 | 0.5 | CLP |  | sub052 | 1 | 10.9 |
| sub25 | 1 | 10.2 | 9.9 | 0.5 | CLP |  | sub053 | 2 | 11.5 |
| sub26 | 2 | 6.0 | 5.7 | 0.5 | CLP |  | sub054 | 1 | 7.4 |
| sub27 | 2 | 10.5 | 10.0 | 0.4 | CP |  | sub055 | 2 | 9.3 |
| sub28 | 2 | 8.8 | 8.3 | 0.3 | CLP |  | sub056 | 1 | 9.0 |

Note: In sex columns, 1 represents male, 2 represents female. NSCLP: nonsyndromic cleft lip and palate, HC: healthy comparison, CLP: cleft lip and palate, CP: cleft palate.

Table S2 Between-group differences in Φ_norm_ and Φ for a range of ks.

| k | Φnorm(NSCLP-HC) | | Φ(NSCLP-HC) | |  |
| --- | --- | --- | --- | --- | --- |
|  | t | p | t | p |  |
| 3 | -1.737 | 0.088 | 1.233 | 0.223 |  |
|  |  |  |  |  |  |
| 4 | -1.728 | 0.090 | 2.794 | 0.007 |  |
|  |  |  |  |  |  |
| 5 | -3.433 | 0.001 | 4.116 | 0.000 |  |
|  |  |  |  |  |  |
| 6 | -4.309 | 0.000 | 5.482 | 0.000 |  |
|  |  |  |  |  |  |
| 7 | -5.057 | 0.000 | 5.666 | 0.000 |  |
|  |  |  |  |  |  |
| 8 | -5.045 | 0.000 | 5.699 | 0.000 |  |
|  |  |  |  |  |  |
| 9 | -5.716 | 0.000 | 5.803 | 0.000 |  |
|  |  |  |  |  |  |
| 10 | -5.871 | 0.000 | 5.857 | 0.000 |  |
|  |  |  |  |  |  |
| 11 | -5.422 | 0.000 | 5.281 | 0.000 |  |
|  |  |  |  |  |  |
| 12 | -4.896 | 0.000 | 5.415 | 0.000 |  |
|  |  |  |  |  |  |
| 13 | -5.223 | 0.000 | 5.993 | 0.000 |  |
|  |  |  |  |  |  |
| 14 | -5.190 | 0.000 | 6.089 | 0.000 |  |
|  |  |  |  |  |  |
| 15 | -4.945 | 0.000 | 5.918 | 0.000 |  |
|  |  |  |  |  |  |
| 16 | -4.623 | 0.000 | 5.768 | 0.000 |  |
|  |  |  |  |  |  |
| 17 | -3.772 | 0.000 | 5.817 | 0.000 |  |
|  |  |  |  |  |  |
| 18 | - | - | 5.576 | 0.000 |  |
|  |  |  |  |  |  |

Φ_norm_: normalized rich-club coefficients, Φ: rich-club coefficients, k: degree, NSCLP: nonsyndromic cleft lip and palate, HC: healthy comparison.


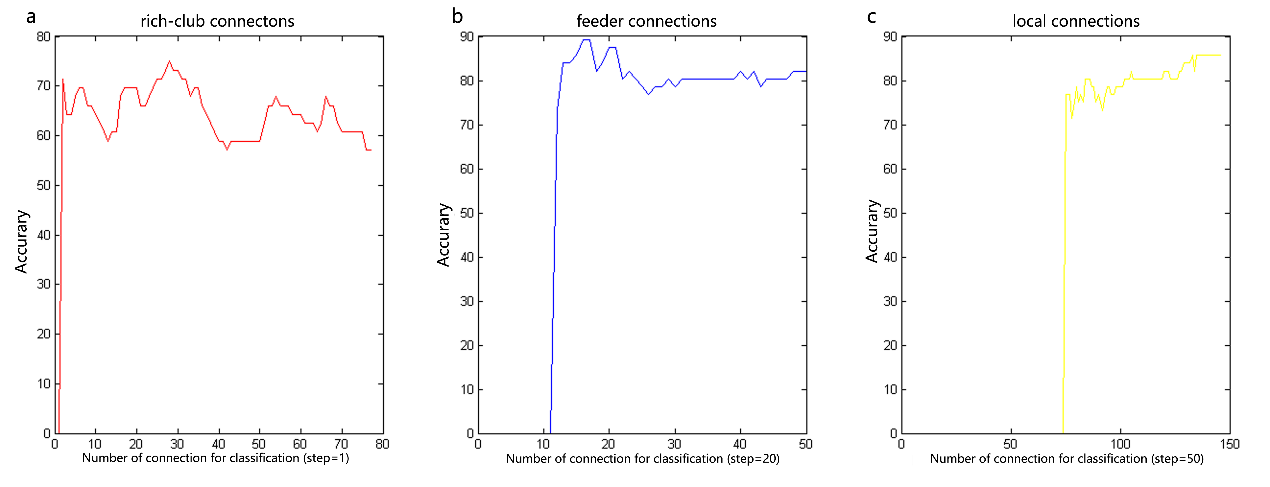


**Figure S1** Predictive accuracy as a function of the number of connections used in the classification process. The connections were ranked according to F scores in descending order. A: rich-club connections (red), B: feeder connection (blue) C: local connections (yellow).


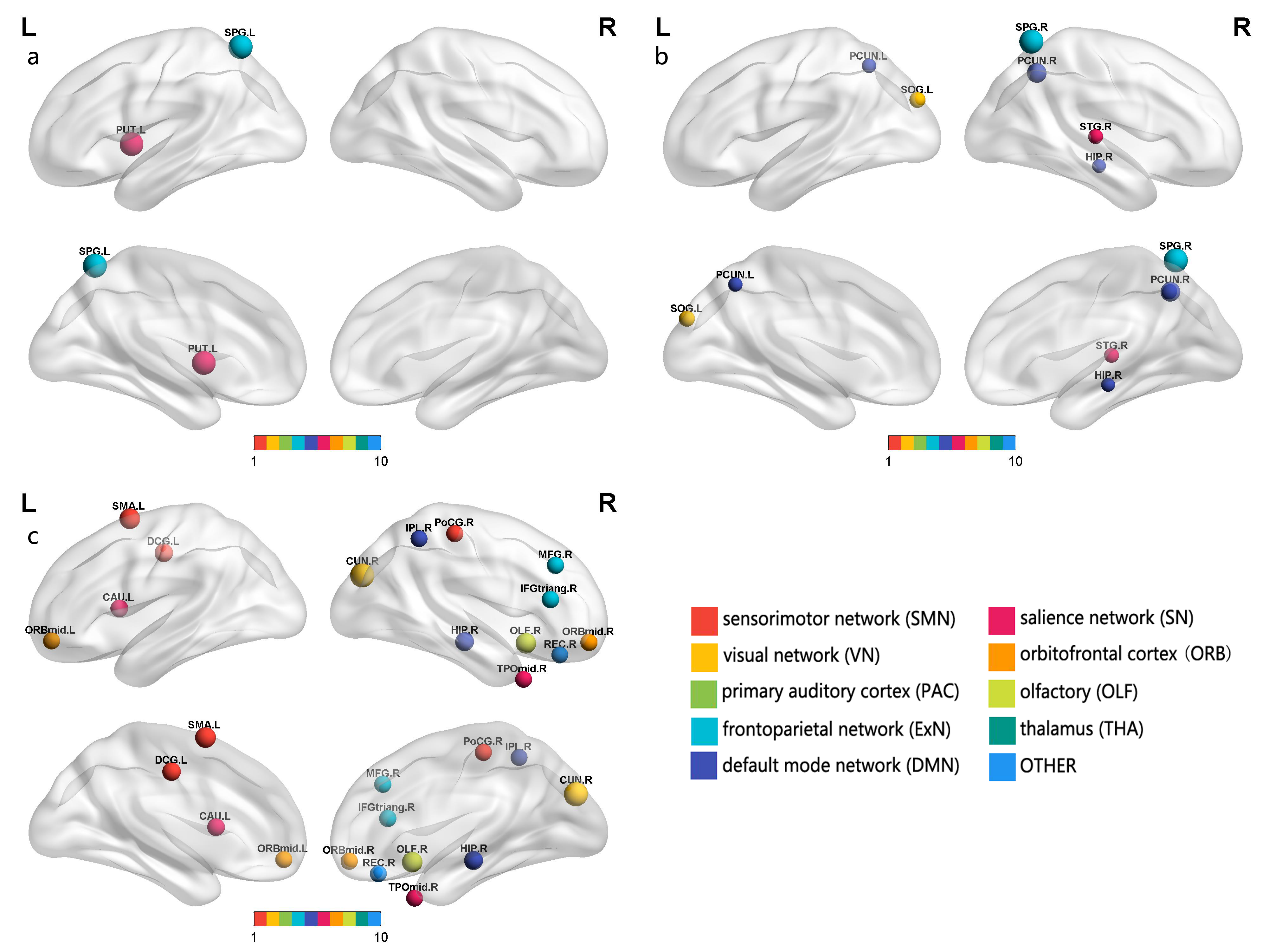


**Figure S2** The distribution of the weighted regions for rich-club (A), feeder (B), and local (C) connections. The size of the balls indicates the classification weights, and the colors represent the intrinsic connectivity networks.


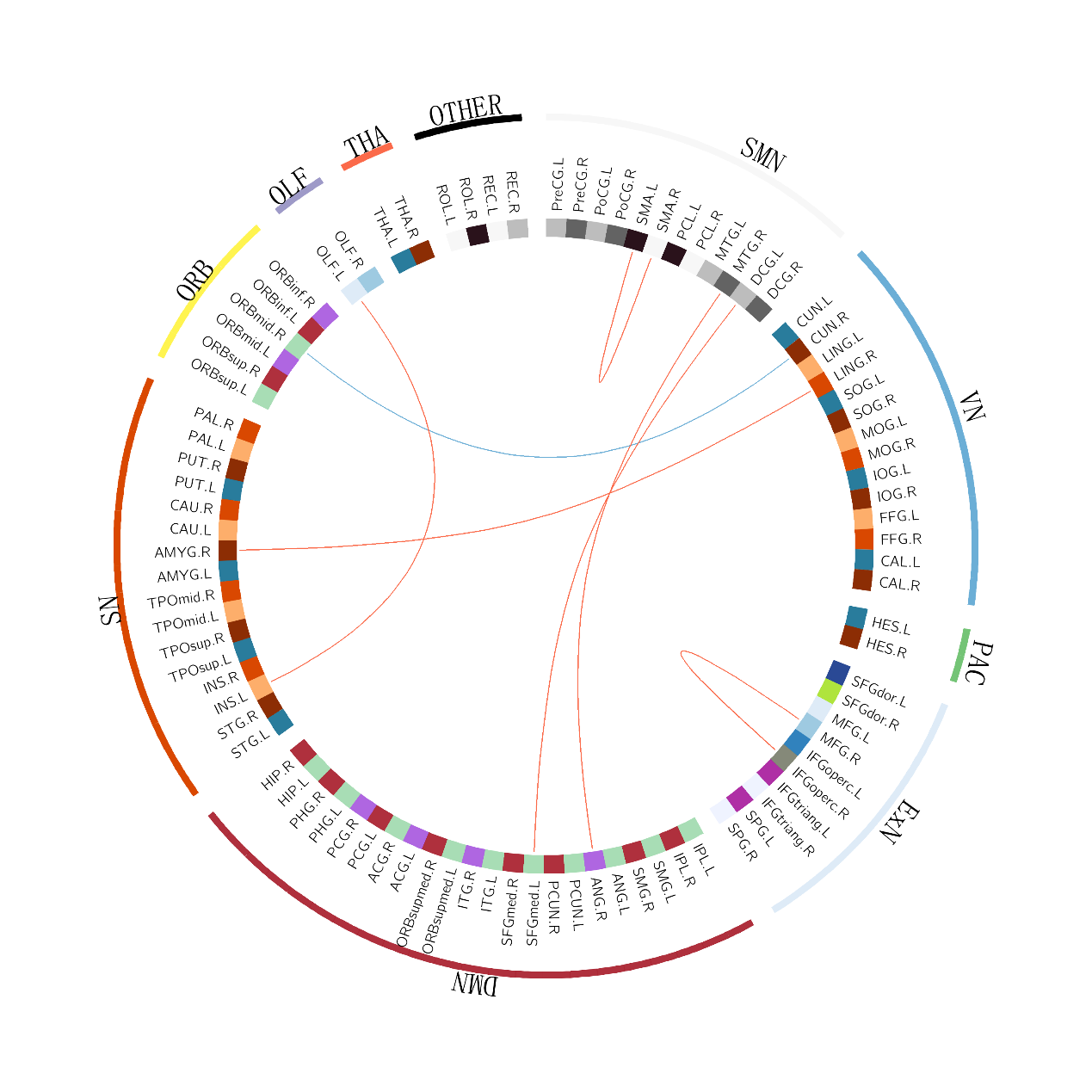


**Figure S3** The connections significantly correlated with the CLCDS scores. The red lines represent positive correlations, and the blue lines represent negative correlations. SMN: sensorimotor network, VN: visual network, ExN: external frontoparietal network, DMN: default mode network, SN: salience network, ORB: orbitofrontal cortex, PAC: primary auditory cortex, TAH: thalamus, OLF: olfactory cortex.

**Note:**

Table S_rich_t.xlsx and table S_rich_p.xlsx recorded the t and p values of the significant between-group differences for the rich-club connections, respectively. Table S_feeder_t.xlsx and table S_feeder_p.xlsx recorded the ones for the feeder connections, respectively.

Table S_local_t.xlsx and table S_local_p.xlsx recorded the ones for the feeder connections, respectively.

Table S_consensus feature.xlsx recorded the consensus features and their classification weights for the three types of structural connections, respectively.

The connection_correlation_result.xlsx recorded the structural connectivity value (FA) significantly correlated with the CLCDS score.
