# Supplementary figures and images for: Random Network and Non-rich-club Organization Tendency in Children With Non-syndromic Cleft Lip and Palate After Articulation Rehabilitation: A Diffusion Study
Source: Front Neurol. 2022 Feb 2;13:790607. doi: 10.3389/fneur.2022.790607 (PMC8847279; doi:10.3389/fneur.2022.790607)

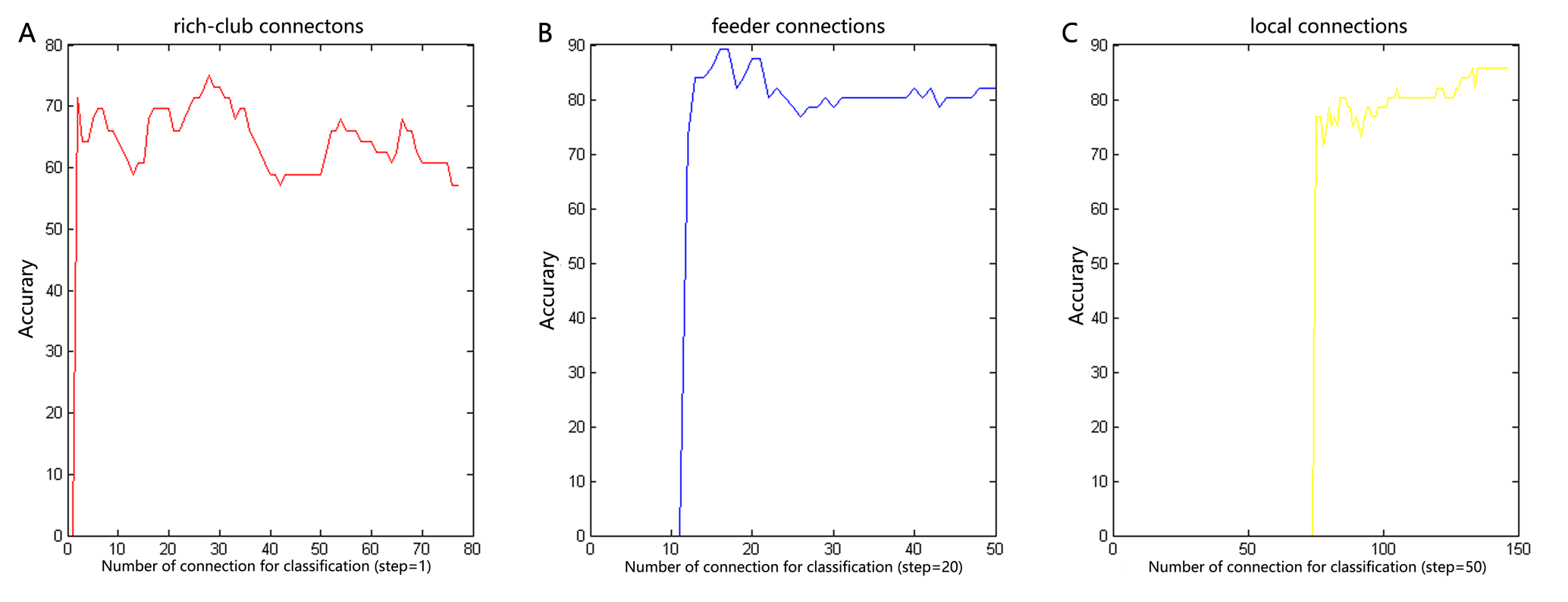

Supplement: Supplementary file 1 [file Data_Sheet_1.zip › supplement/Figure s1.tif]

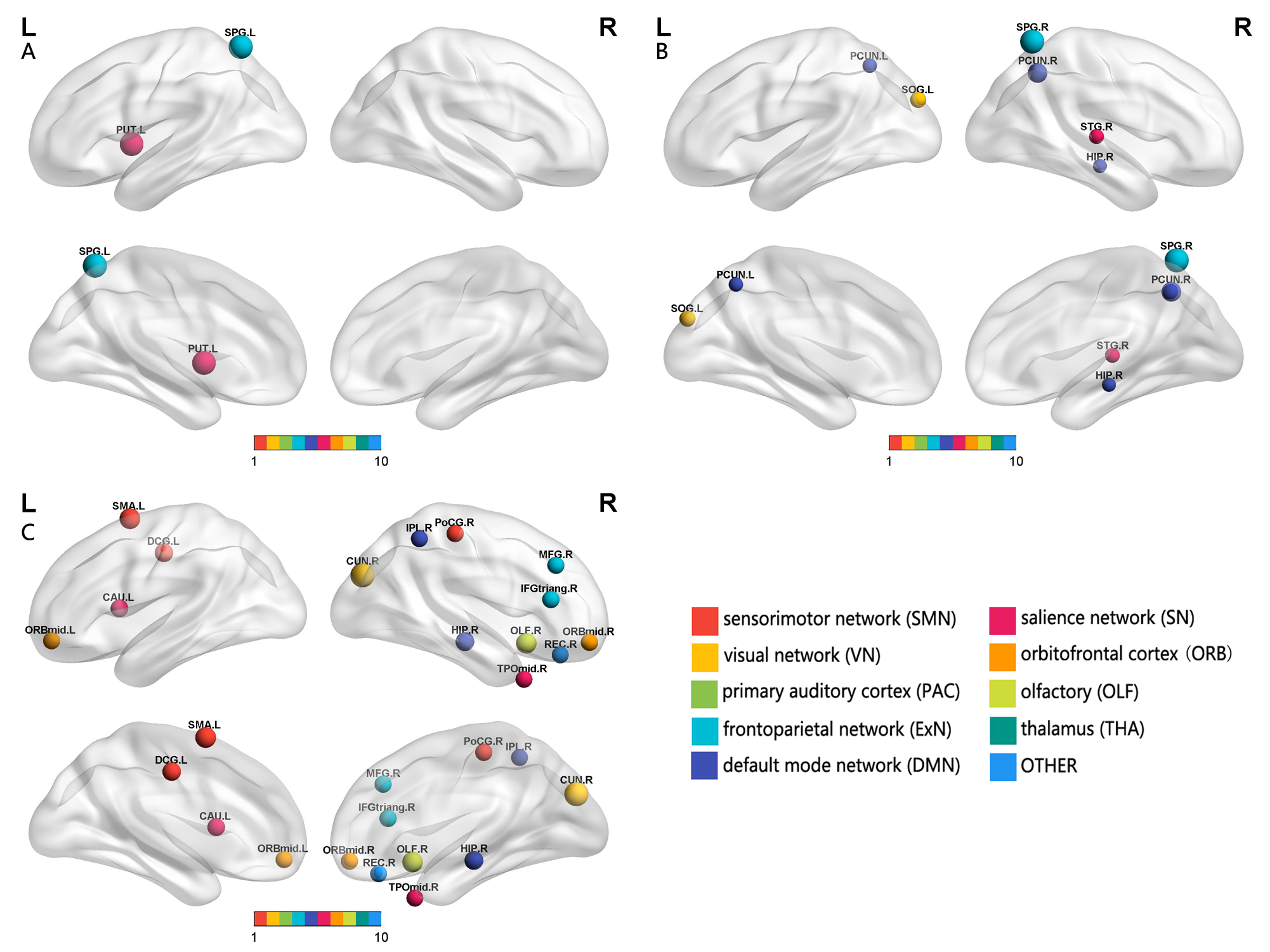

Supplement: Supplementary file 1 [file Data_Sheet_1.zip › supplement/figure s2.tif]

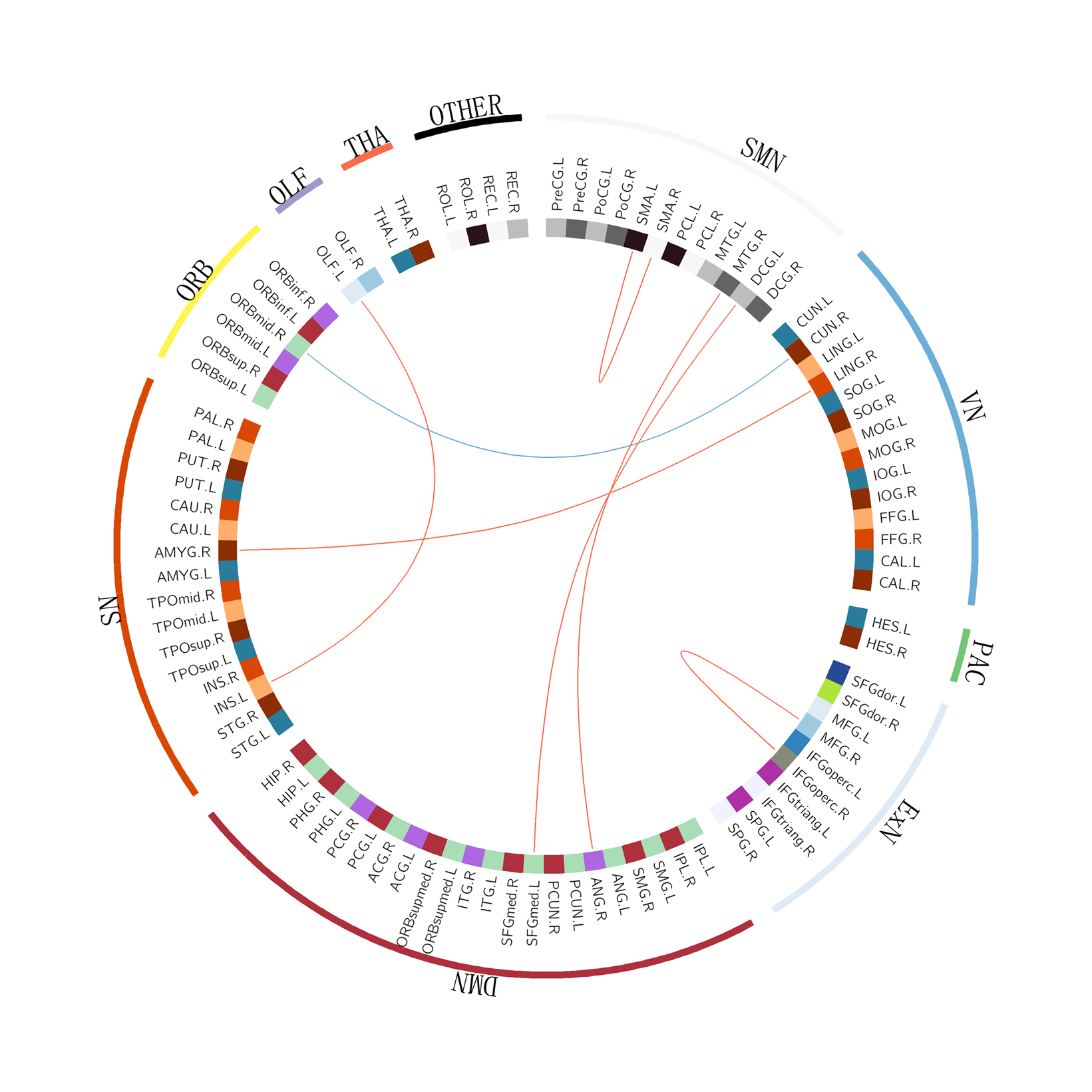

Supplement: Supplementary file 1 [file Data_Sheet_1.zip › supplement/figure s3.tif]
